# Plus Ça Change: Gene Enhancers Upset Evolutionary Assumption

**DOI:** 10.1371/journal.pbio.0030138

**Published:** 2005-03-15

A standard evolutionary assumption is that the DNA of closely related species should be more similar in both structure and function than that of more distantly related ones. This parsimonious rule of thumb holds true across wide expanses of time and among widely divergent species, but it has exceptions. In this issue, Michael Ludwig and colleagues show that one exception is in an enhancer region of a key developmental gene in fruitflies of the genus Drosophila. Here, the enhancer is functionally similar enough in two species that diverged 60 million years ago that switching them produces normal development, but different enough in two species separated by only 10 million years that exchanging them between the two flies aborts development.

The gene in question is *even-skipped*, a patterning gene that creates seven transverse stripes along the anterior–posterior axis in the fruitfly embryo. Its expression is regulated by five enhancing elements located upstream from the promoter. The best characterized of these, the stripe 2 enhancer (S2E), binds five different transcription factors at multiple locations.

Ludwig et al. deleted S2E in D. melanogaster, and then added back S2E from one of four Drosophila species: D. melanogaster itself; D. yakuba or D. erecta, both of which have been separated from D. melanogaster for 10–12 million years; or D. pseudoobscura, which split from the D. melanogaster line 40–60 million years ago. Despite serving identical functional roles in each species, the structures of these enhancers differ, with large deletions and insertions between transcription factor binding sites, as well as other changes. Nonetheless, within each species, the spatiotemporal pattern of expression induced by S2E is essentially identical, suggesting that, despite their structural differences, they might be functionally interchangeable.

Since loss of S2E is lethal, the viability of the embryos that resulted from these experiments gives a measure of each enhancer's ability to function in its new environment. The authors found that while viability of D. melanogaster with the D. pseudoobscura S2E was identical to that with D. melanogaster's own, viability with S2E from the more closely related D. erecta was almost zero, essentially the same as not having the enhancer at all. S2E from D. yakuba impaired the viability of D. melanogaster as well, although not as much as that from D. erecta. Viability was closely correlated with the level of stripe 2 expression induced by each S2E, with very low levels induced by the D. erecta enhancer and normal levels by that of D. melanogaster and D. pseudoobscura.

Why did the D. erecta enhancer fail to respond in the D. melanogaster environment? Ludwig et al. suggest it may be due to a change in the sensitivity of the “set point” in D. erecta's enhancer, which acts like an on–off switch governing gene expression, making the enhancer unresponsive to the gradients of transcription factors found in D. melanogaster. This change may be due to relatively small differences in the two species' enhancers that have accumulated since their evolutionary split.

The results of this study indicate an important caveat about interpreting the evolution of gene regulatory regions. As a complex functional unit that integrates a host of signals, the S2E is likely to be under strong stabilizing selection, maintaining its output within narrow limits. Thus, the phenotypic result of the enhancer—the location and timing of stripe formation it induces in its native environment—remains conserved among the four species. However, unlike an enzyme or structural protein, in which structural changes are tightly constrained by their effects on function, the structure of any particular enhancer need not be so rigidly preserved. As long as the consequences of change in one region, such as loss of a transcription factor binding site, are matched by compensatory changes in another, such as gain of one, or, as Ludwig et al. speculate, by complementary changes in genetic background, the final output of the enhancer can remain the same. Thus, the utility of structural similarities in understanding evolutionary relationships is likely to be less for gene regulatory regions than for structural genes or the proteins they encode.

